# Characterizing the Gut Microbiota of Eurasian Otter (*Lutra lutra chinensis*) and Snub-Nosed Monkey (*Rhinopithecus roxellana*) to Enhance Conservation Practices in the Foping National Nature Reserve of China

**DOI:** 10.3390/ani12223097

**Published:** 2022-11-10

**Authors:** Dapeng Zhu, Tongtong Xie, Ruifang Du, Long Guo

**Affiliations:** 1 State Key Laboratory of Grassland Agro-Ecosystem, Lanzhou 730020, China; 2 College of Pastoral Agriculture Science and Technology, Lanzhou University, Lanzhou 730020, China; 3 Foping National Nature Reserve, Hanzhong 723000, China

**Keywords:** gut microbiota, Eurasian otter, snub-nosed monkey, Foping National Nature Reserve, soil

## Abstract

**Simple Summary:**

This study analyzed the relationship between soil microbes and the microbial diversity and structure of the distal gut of the terrestrial golden snub-nosed monkey and Eurasian otter in the Foping National Nature Reserve (FNNR), Shaanxi Province, China, and provides baseline information regarding the gut microbiota of these wild species.

**Abstract:**

Understanding the interaction between the microbial composition in the habitat and the gut of wildlife will contribute to conservation efforts since changes in the gut microbiome have been proven to influence the healthy and nutritional status of the host. This study analyzed the relationship between soil microbes and the microbial diversity and structure of the distal gut of the terrestrial golden snub-nosed monkey and Eurasian otter in the Foping National Nature Reserve (FNNR). A total of 15 otter fecal samples and 18 monkey fecal samples were collected from which 5 and 6 samples, respectively, were randomly selected for microbiome analysis. The remaining samples were used for fecal short-chain fatty acids (SCFAs) analysis. Soil samples from the otter and monkey habitats at each sampling point (eight in total) were also collected for microbiome analysis. The microbial phyla with the greatest relative abundance in soil or animal samples were Proteobacteria (41.2, 32.7, and 73.3% for soil, otters, and monkeys, respectively), Firmicutes (0.4% soil, 30.1% otters, and 14.4% monkeys), Bacteroidota (5.6% soil, 17.0% otters, and 8.3% monkeys), and Acidobacteriota (24.6% soil, 1.7% otters, and 0.1% monkeys). The estimation of alpha diversity indices revealed that the feature, Chao1, and Shannon indices of the soil microbiome were the greatest (*p* < 0.01) among the three groups, followed by those of the otter microbiome and those of the monkey microbiome (*p* < 0.01). Beta diversity analyses confirmed differences in the microbiota of the three types of samples. The determination of SCFA concentration in feces revealed that total volatile fatty acids, acetic acid, and isovaleric acid were greater (*p* < 0.05) in otters than in monkeys, while propionic acid followed the opposite pattern (*p* < 0.05). Correlation analysis of the microbiome and SCFA contents showed that propionic acid was positively correlated with significantly different bacterial groups, while acetic and butyric acid and total volatile acids were negatively correlated. This study confirmed that the fecal microbes of Eurasian otters and golden snub-nosed monkeys in the reserve are related to the soil microbial communities of their habitats, but they have different bacterial community structures and compositions, and there are different SCFA metabolic patterns in the gut of the two animals. The present study will help to improve wildlife protection in the FNNR.

## 1. Introduction

The gut microbiome affects the host’s nutritional intake, development, health, and even behavior, while the diet, physiology, and phylogeny of the host all play a crucial role in shaping the composition and function of mammalian gut microbes [1]. Therefore, it is essential to reveal the characteristics of gut microbial communities, as they are intimately connected to the health of animals [2]. For wildlife, research on the gut microbiota is essential for better protection since gut microbes play a significant role in maintaining the host’s health [3]. A previous study on intraspecific variation in gut microbiota, framed using a comparison of wild and captive animals, provided valuable descriptions of differences in supposed extremes [4]. These nature perturbations are generally attributed to commercial food including manufactured chow and cultivated produce [5]. Due to hosts experiencing a broader range of environments than is usually involved in comparisons of wild and captive animals, a wider comparative method is necessary to provide a more comprehensive and detailed understanding of gut microbial variation [6]. In this study, the fecal microorganisms of otters and snub-nosed monkeys in the wild state and soil microorganisms in their habitats were selected to analyze the gut microbiota characteristics of these two protected species from a wild, natural point of view and eliminate the interference of human factors.

Eurasian otters (*Lutra lutra chinensis*) and snub-nosed monkeys (*Rhinopithecus roxellana*) are high-priority species for targeted conservation efforts in the Foping National Nature Reserve (FNNR). The Eurasian otter belongs to the subfamily Lutrinae and is a smaller type of otter and a top predator in freshwater ecosystems [7]. Although these otters are excellent swimmers, they spend most of their time on land and feed mainly on fish and invertebrates such as crustaceans and mollusks and, sometimes, also consume leaves and grass [8]. As a nonhuman primate, there are five species of snub-nosed monkeys, two of which are distributed in Vietnam and Myanmar, while the remaining three inhabit China [9]. In China, a few provinces are the habitats of snub-nosed monkeys, including Shaanxi, Gansu, Yunnan, and Guizhou provinces. Nevertheless, because of continuous declines in the population resulting from human activity and environmental changes, the snub-nosed monkey is classified as a first-class endangered animal in China, with many organizations committed to conducting studies to conserve this species [10].

As we mentioned earlier, animal hosts’ gut microbiota performs crucial functions that promote health, support nutrition, and underlie natural host behavior [11]. All of these important roles have contributed to raising appeals for microbiome science to be incorporated into conservation biology and wildlife management. However, to the best of our knowledge, no valid information was reported on the gut microbiota of wild otters and monkeys in the FNNR of China. This study focused on the relationship between soil microbes and the microbial diversity and microbiota structure of the distal gut of the typical terrestrial snub-nosed monkey and waterway amphibious otter in the FNNR, Shaanxi Province, China, and provides baseline information regarding the gut microbiota of these species. Our team has worked at FNNR for approximately 10 years, and we could collect fresh fecal samples that were able to link with individual animals. Thus, we hypothesize that otters and monkeys share commonalities with their habitat soil microorganisms and each has its own gut microbiota characteristics. Study on gut microbiota conditions is vital for protection, and here, by elucidating the baseline characteristics of the gut microbiota of otters and monkeys, we aimed to improve wildlife protection in the FNNR. As far as we know, the present research was the first observation of the gut microbiota characteristics of wild otters and monkeys.

## 2. Materials and Methods

### 2.1. Overview of Collected Samples

The FNNR is currently the nature reserve with the most complete preservation of virgin forest with the least human disturbance and one of the most critical areas for biodiversity conservation in the Qinling Mountains [12]. The FNNR is located at the southern foot of the middle section of the Qinling Mountains, Shaanxi Province, in central and western China, with a geographic range between 107°41’–107°55’ E and 33°33’–33°46’ N, and 980–2904 m above sea level, with a total area of 29240 hm2. Located in the transition zone to the warm temperate zone, the FNNR is a national nature reserve that is rich in animal and plant resources and has a wide variety of rare and endangered animals, including pandas, Eurasian otters, and snub-nosed monkeys.

**Figure 1:**
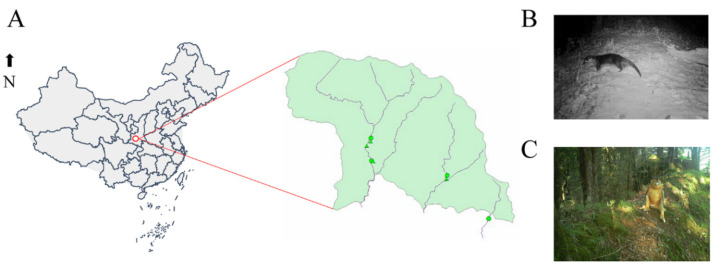
Map of Foping National Nature Reserve, China, highlighting the three field sites, including the sample collection locations (triangle: Otters; circle: Monkeys), separated by the river (blue line) (**A**); and photo of the study species, Eurasian otter (*Lutra lutra chinensis*) (**B**) and golden snub-nosed monkey (*Rhinopithecus roxellanae*) (**C**). Photos by Dapeng Zhu.

Three river stretches were chosen for sampling (Figure 1A), which are home to endangered animals, including Eurasian otters (Figure 1B) and snub-nosed monkeys (Figure 1C). The selected river stretches were adequately far apart to ensure use by different otters. We chose six to eight otter marking places to collect the fecal samples at each river stretch. In January 2022, these sampling points were checked at sunrise on 5 consecutive days to ensure that fecal samples were collected within 5 h of deposition by the otter or monkey. Otter feces are easily recognizable to the trained eye and are usually deposited by otters in prominent places as a marking activity for territorial defense. Otter feces collected from the same river stretches might be from the same individual, but feces from different river stretches are most likely not. The monkeys’ habitat is distributed in the woods around the otter habitat, and their feces were also collected. Soil samples (approximately 10 g) from the otter and monkey habitats at each sampling point (8 in total) were also collected for microbiome analysis. A total of 15 otter fecal samples and 18 monkey fecal samples were collected from which 5 and 6 samples, respectively, were randomly selected for microbiome analysis. The remaining samples were used for fecal short-chain fatty acid analysis.

### 2.2. Microbial Community Analysis

The protocol for the extraction of microbial DNA was employed according to a previous study in the literature [13]. The obtained samples (*n* = 19), including soil (8), otters (5), and monkeys (6), were analyzed by 16S rDNA sequencing using the Illumina HiSeq 2500 platform (BaiMike Biotechnology Co., Ltd., Beijing, China). A sequencing library (SMRT Bell) was constructed using PCR amplification, purification, quantification, and homogenization. We used the Smart Link tool (PacBio) to obtain the CCS sequence. CCS was identified by barcodes using Limav 1.7.0 software to obtain RAW-CCS sequence data. The primer sequences were identified and removed using Cutadapt 1.9.1 software, and we conducted length filtering to obtain clean CCS sequences without primer sequences. Finally, the UCHIME v4.2 software was used to remove chimeras to obtain an effective CCS sequence. The operational taxonomic units (OTUs) were used to cluster and divide the high-quality sequences with 97% sequence similarity. The samples were analyzed by taxonomy based on OTUs, and original data and community structure maps were obtained at the phylum, class, order, family, and genus levels. The indices, including feature, Chao1, Shannon, and Simpson, were obtained, and principal coordinate analysis (PCoA) was implemented to compare species diversity and richness among different samples of soil and fecal samples of otters and monkeys.

### 2.3. Short-Chain Fatty Acid Determination

The extraction and determination of short-chain fatty acids (SCFAs) were performed according to the literature [14]. After thawing at 4 °C, 5 mL of the diluent was added to fecal samples and then centrifugated at 2500× *g* for 5 min. Then, the supernatant (approximately 5 mL) was mixed with 1 mL of 25% metaphosphoric acid in a centrifuge tube. The mixture was placed at 4 °C for 3 h. After that, the mixture was centrifuged at 3000× *g* for 10 min. Then, the supernatant (approximately 2 mL) was centrifuged at 12,000× *g* at 4 °C for 15 min and filtered through a 0.45 μm organic membrane. One milliliter of the filtrate and 200 μL of 1% crotonic acid were mixed and the mixture was used for gas chromatography (GC-2010 PLUS, Shimadzu, Japan) to determine the SCFAs content in fecal samples.

### 2.4. Statistical Analysis

The results were analyzed by a completely randomized design. All statistical analyses were performed by the GLM (general linear model) procedure of the Statistical Analysis System (SAS Inst. Inc., Cary, NC, USA). One-way ANOVA was used to analyze the data of all indices. The statistical model was as follows:

Yi = μ + Ai + Bi,

in which Yi, μ, Ai, and Bi represent the dependent variable, overall mean, species effect, and the error term, respectively.

Multiple comparisons of means were performed by Duncan’s test. A significant difference was defined as *p* < 0.05 and trends were defined as 0.05 < *p* < 0.10.

## 3. Results

### 3.1. Sequencing Statistics

**Figure 2:**
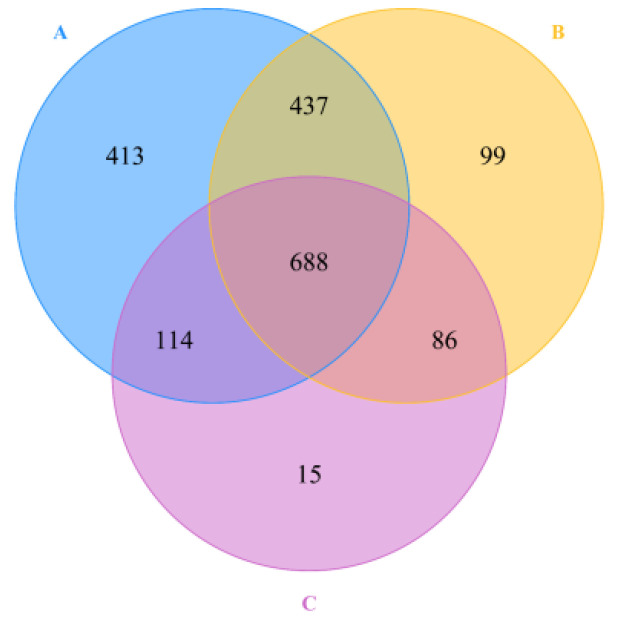
Venn diagram of OTUs between the habitat soil microbiome (**A**) and fecal microbiome of Eurasian otters (**B**) and golden snub-nosed monkeys (**C**).

A total of 1,411,944 (average length of 421 bp) chimera-free high-quality sequences were recovered, with an average of 74,313 sequences per sample. These sequences were assigned to a total of 1852 OTUs at a 97% sequence identity level, which was sorted from 19 samples. Each sample had 897 ± 401 OTUs on average, ranging from 274 to 1400. The shared number of OTUs (688 OTUs) accounted for 37.2% of the total OTUs and was the highest, followed by the number of unique OTUs in the soil (413 OTUs, 22.3% of the total OTUs), otters (99 OTUs, 5.4%), and monkeys (15 OTUs, 0.8% of the total OTUs) (Figure 2).

### 3.2. Composition of Fecal Microbial Communities

**Figure 3:**
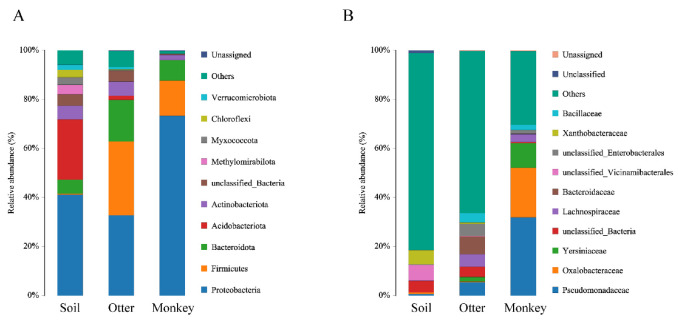
Composition of the microbial community at the genus level between groups (**A**) and individual samples (**B**).

The microbial phyla with the greatest relative abundance in soil or animal samples were Proteobacteria (41.2, 32.7, and 73.3% relative abundance for soil, otters, and monkeys, respectively), Firmicutes (0.4% soil, 30.1% otters, and 14.4% monkeys), Bacteroidota (5.6% soil, 17.0% otters, and 8.3% monkeys), and Acidobacteriota (24.6% soil, 1.7% otters, and 0.1% monkeys) (Figure 3A). The composition of microbial families was different among the three types of samples (Figure 3B). The microbial families of the soil (18.6%) were the least enriched at the family level, followed by otters (33.6%) and monkeys (69.6%). The three most abundant families in soil were unclassified_Vicinamibacterales, Xanthobacteraceae, and unclassified_Bacteria, while Bacteroidaceae, Pseudomonadaceae, and Lachnospiraceae were found in otters, and Pseudomonadaceae, Oxalobacteraceae, and Yersiniaceae were found in monkeys.

### 3.3. Diversity

**Figure 4:**
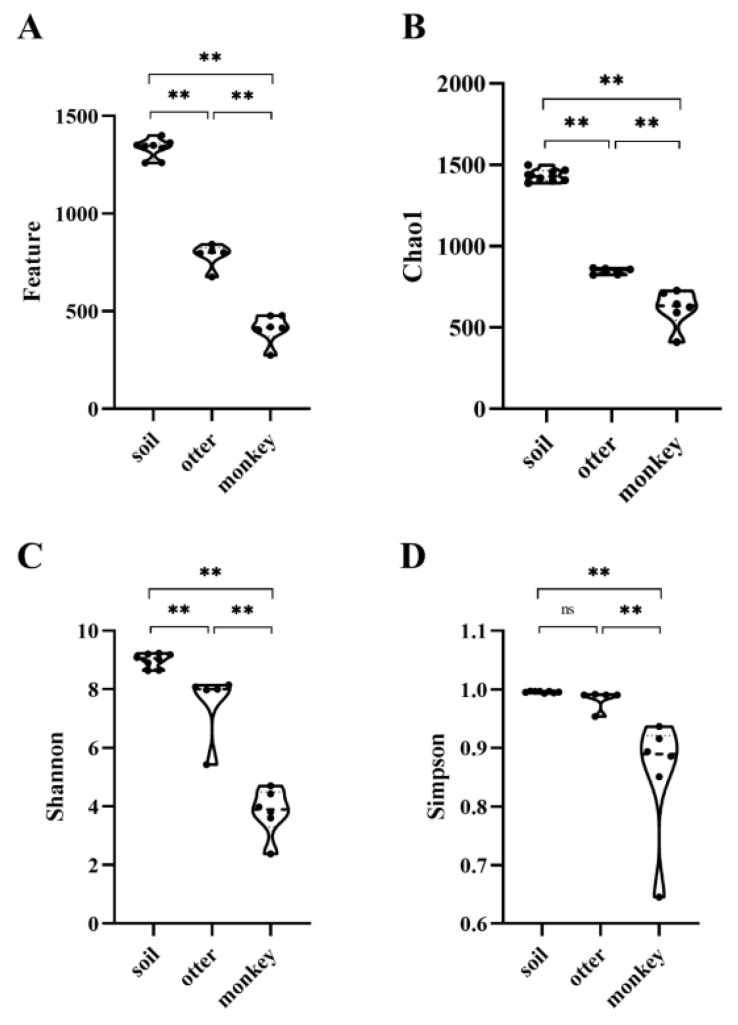
Alpha diversity, including the feature (**A**), Chao1 (**B**), Shannon (**C**), and Simpson (**D**) indices, between the habitat soil microbiome and fecal microbiomes of Eurasian otters and golden snub-nosed monkeys. ** *p* < 0.01.

Estimation of alpha diversity indices revealed that the feature (Figure 4A), Chao1 (Figure 4B), and Shannon (Figure 4C) indices of the soil microbiome were the greatest (*p* < 0.01) among the three groups, followed by those of the otter microbiome, and those of the monkey microbiome were the lowest (*p* < 0.01). The Simpson indices for the soil and otter microbiomes were not significantly different (*p* > 0.05) but they were significantly higher (*p* < 0.01) than that of monkeys.

**Figure 5:**
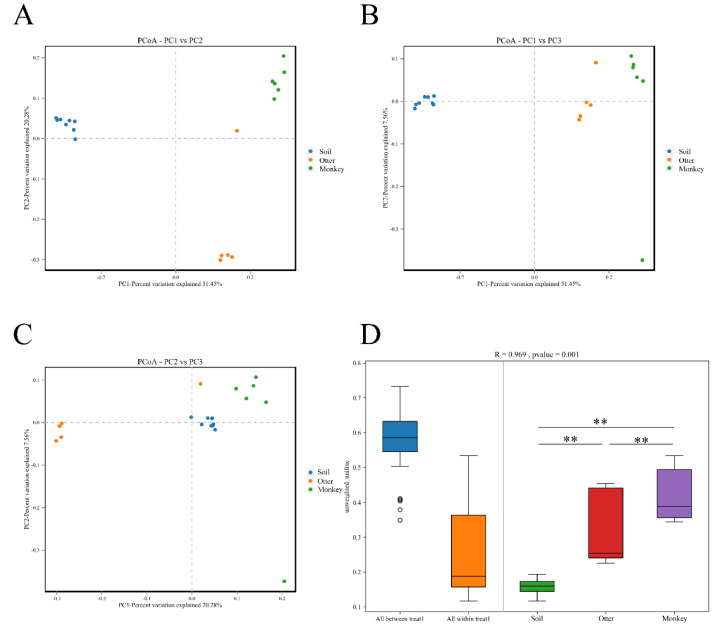
Beta diversity (unweighted UniFrac distances) of the habitat soil microbiome and gut microbes of Eurasian otters and golden snub-nosed monkeys. (**A**–**C**) represented PcoA PC1 vs. PC 2, PC1 vs. PC3, and PC2 vs. PC3, respectively. (**D**) Box diagram of PERMANOVA/Anosim. ** *p* < 0.01.

The beta diversity analyses based on unweighted UniFrac distances confirmed differences in the three groups of microbiota samples (Figure 5A–C). The comparisons of UniFrac distances among sample groups further highlight group-level variations. Overall, there were significant differences in mean pairwise distances between the three study groups (R = 0.969, *p* < 0.001), with the post-hoc tests confirming that the distances of monkeys were greatest among all three samples groups, followed by otters and soil (Figure 5D).

### 3.4. Difference Analysis

**Figure 6:**
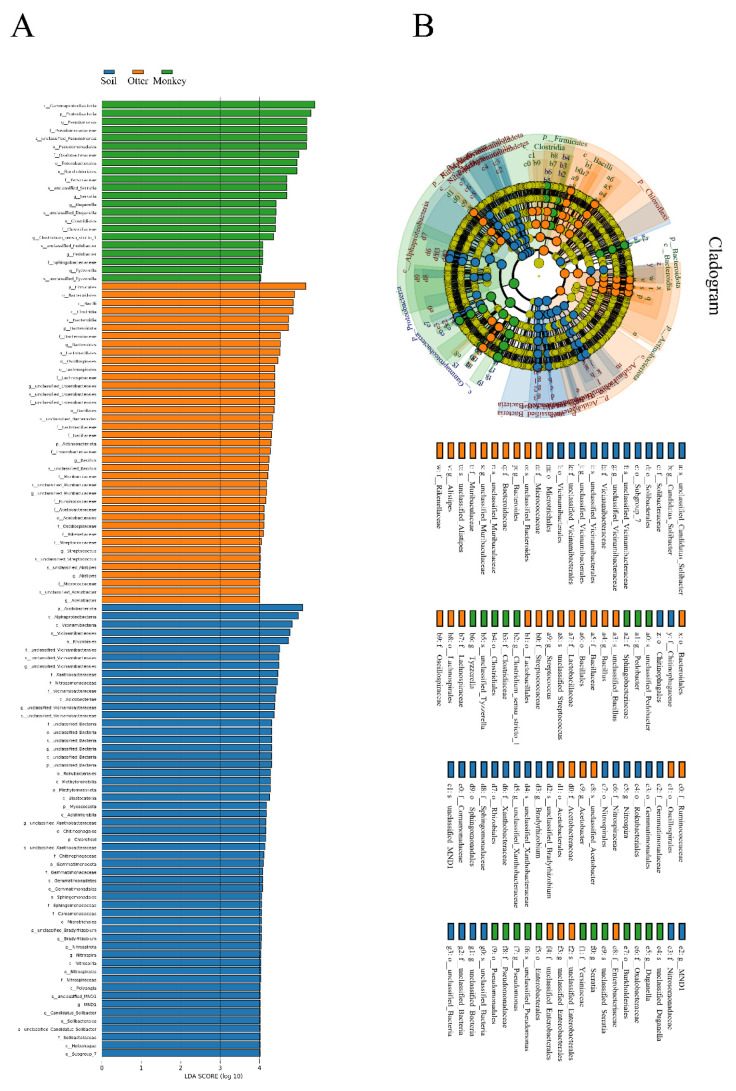
Histogram of the linear discriminant analysis (LDA) effect size (**A**) and cladogram (**B**) for genera differentially abundant between the habitat soil microbiome and gut microbes of Eurasian otters and golden snub-nosed monkeys. Only the genera with a linear discriminant analysis significance threshold >2 are shown.

The linear discriminant analysis effect size (LEfSe) (Figure 6A) showed that 116 genera were identified as discriminative features between the samples from the soil, otter, and monkey groups. The number of genera enriched in the soil, otter, and monkey samples was 55, 39, and 22, respectively. The microbial evolutionary branches at the genus level for the three sample groups were intricate and defy categorical regularity (Figure 6B).

### 3.5. Prediction of Microbial Phenotype

**Figure 7:**
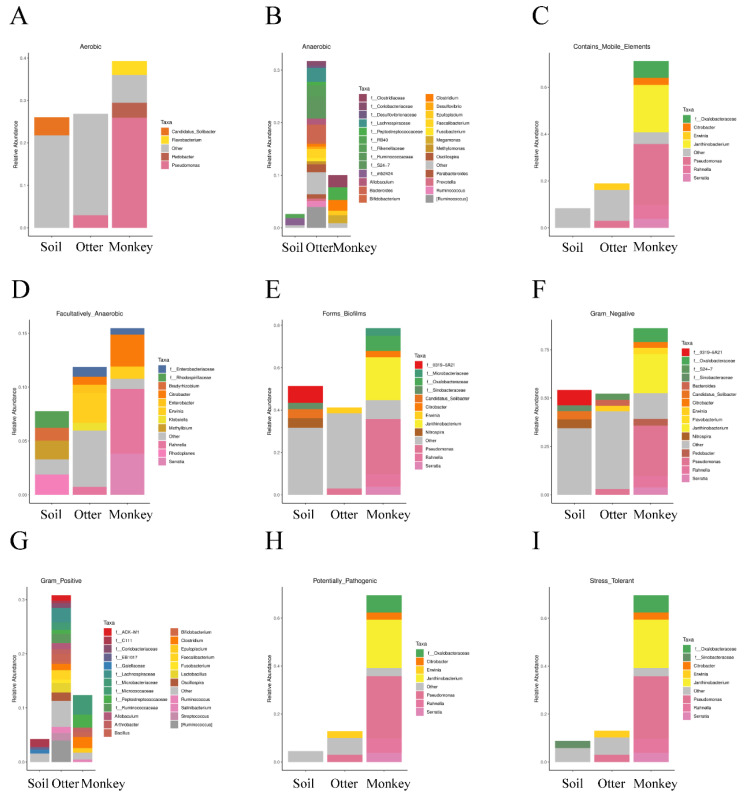
Microbiota phenotype prediction of the habitat soil microbiome and gut microbiomes of Eurasian otters and golden snub-nosed monkeys: “aerobic” (**A**), “anaerobic” (**B**), “contains mobile elements” (**C**), “facultatively anaerobic” (**D**), “forms biofilms” (**E**), “Gram-negative” (**F**), “Gram-positive” (**G**), “potentially pathogenic” (**H**), and “stress-tolerant” (**I**) phenotypes.

According to the findings of the microbial phenotypic prediction (Figure 7), the “anaerobic” (Figure 7B) and “Gram-positive” (Figure 7G) phenotypes were found to have the largest abundances and number of microbial groups in the otter fecal samples, whereas other microbial phenotypes were most abundant in monkey guts, namely, “aerobic” (Figure 7A), “contains mobile elements” (Figure 7C), “facultatively anaerobic” (Figure 7D), “forms biofilms” (Figure 7E), “Gram-negative” (Figure 7F), “potentially pathogenic” (Figure 7H), and “stress-tolerant” (Figure 7I).

### 3.6. Concentration of Short-Chain Fatty Acids

**Figure 8:**
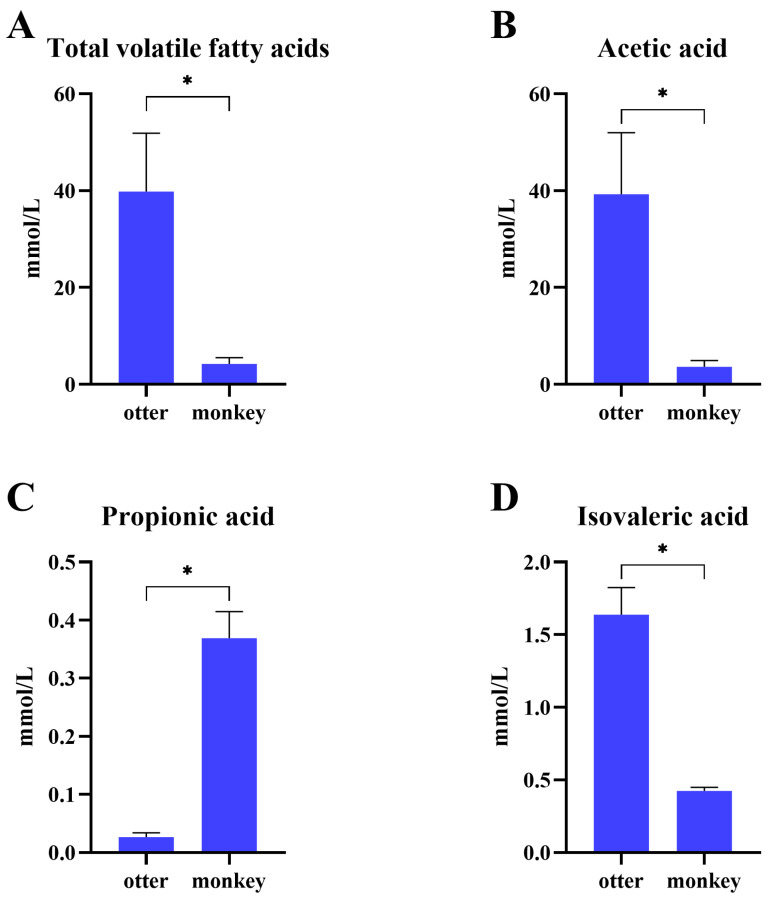
Short-chain fatty acid concentrations, including total volatile fatty acids (**A**), acetic acid (**B**), propionic acid (**C**), and isovaleric acids (**D**) in feces from Eurasian otters and golden snub-nosed monkeys. * *p* < 0.05.

Analysis of the SCFA concentration in feces (Figure 8) revealed that the total volatile fatty acids, acetic acid, and isovaleric acid concentrations were greater (*p* < 0.05) in otters than in monkeys, while that of propionic acid showed the opposite pattern (*p* < 0.05).

### 3.7. Correlations between the Fecal Microbiota and Short-Chain Fatty Acid Profile

**Figure 9:**
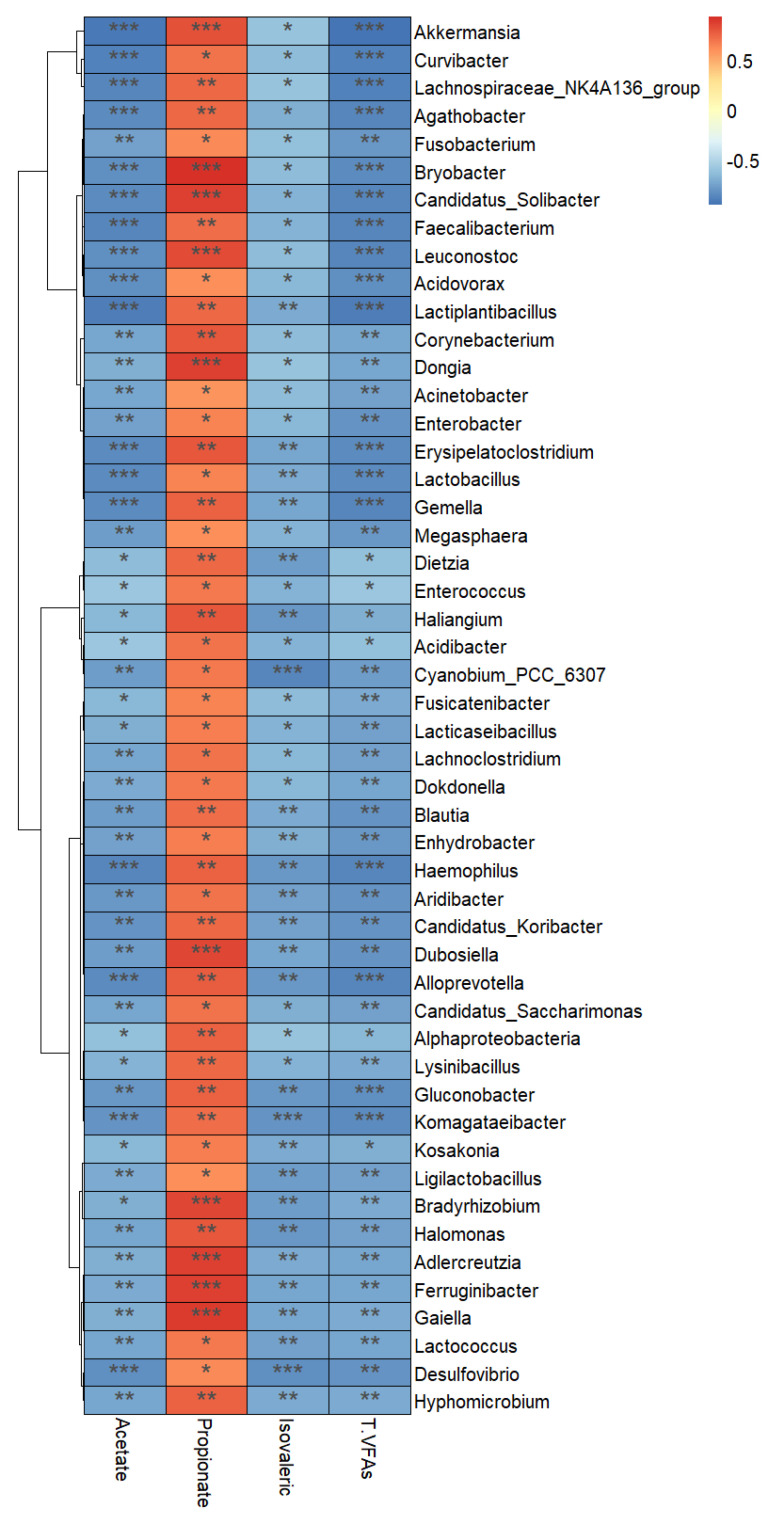
Correlation heatmap between changed fecal microbiota and altered short-chain fatty acids in Eurasian otters and golden snub-nosed monkeys. The correlation coefficients with a statistical *p* value <0.05 and an absolute value >0.7 were used to construct the network graph. * *p* < 0.05, ** *p* < 0.01, *** *p* < 0.001.

Correlation analysis was performed using the data regarding changed genera obtained from the LEfSe and significantly different SCFAs in the feces of the otter and monkey groups (Figure 9). The results indicated that the top 10 bacterial taxa that showed the strongest negative correlations with acetic acid, isovaleric acid, and total SCFAs were Akkermansia, Curvibacter, Lachnospiraceae_NK4A136_group, Agathobacter, Fusobacterium, Bryobacter, Candidatus_Solibacter, Faecalibacterium, Leuconostoc, and Acidovorax. The top 50 bacteria listed were all positively correlated with propionic acid.

## 4. Discussion

The two animals, Eurasian Otter (*Lutra lutra chinensis*) and the golden snub-nosed monkey (*Rhinopithecus roxellana*), are both protected under the Law of the People’s Republic of China on the Protection of Wildlife, are considered endangered, and may be keystone species within FNNR, making them a high-priority target for conservation efforts. Here, we used microbial 16S sequencing technology, combined with the gas chromatography technique, to characterize and analyze the distal gut microbiotas and their fermentation metabolites of Eurasian otters and snub-nosed monkeys and the microbiota structure of their habitats. Through fecal and soil sampling from multiple sites representing the otter’s and monkey’s natural range in the FNNR, we found that (1) soil microbes and animal microbes are “heterogeneous” and share common microbes, (2) there is a profound difference in the gut microbiome composition between the Eurasian otter and golden snub-nosed monkey within the same natural region, and (3) different animal species have different metabolic patterns of intestinal SCFAs. A previous study showed host ‘group signatures’ in microbiota, always attributed to microbial social transmission [15]. Our findings direct attention to the potential effects of microbial exchange between animal hosts and the environment in mediating significant variation of the interpopulation.

The composition and function of microbiome community are affected by various factors, including host phylogeny [16], diet [17], and external environmental variables [18]. Broad patterns in microbiome composition are often associated with animal health and performance. In our study, the microbiota from the soil and feces of otters and monkeys showed a similar structure in terms of relative distribution. For example, all three types of samples harbored similar dominant families (*Pseudomonadaceae*), but the proportions of the *Proteobacteria* and *Firmicutes* phyla differed. In wild snub-nosed monkeys, the relative abundance of *Bacteroidetes*, *Proteobacteria,* and *Firmicutes* indicated they were the top three dominant bacteria [19], which is consistent with the present study. The significantly enriched abundance of *Proteobacteria* genera (e.g., *Pseudomonas*, *Comamonas*, and *Acinetobacter*) might be a response to bamboo shoot intake in the diet of monkeys. Moreover, the significantly enriched abundance of specific *Firmicutes* might be involved in the digestion of high-carbohydrate foods. We have also shown that the otter fecal microbiome is composed mainly of *Firmicutes*, *Proteobacteria*, and *Bacteroidota*. This gut bacterial structure is similar to that of the American mink and Eurasian river otter gut microbiota [20]. In wild Eurasian otter feces, *Bacteroides* were found to be more abundant [21]. *Bacteroides*, which are known as protein and fat degraders, were reported to be the dominant genus in the gut microbiota of spotted hyenas [22] and Amur tigers [23]. In the present study, *Bacteroidaceae* was also more abundant in the otter feces, indicating the otters’ carnivorous (mainly fish) diet. *Enterobacter* and *Pseudomonas* have also been reported to be highly abundant in the fecal microflora of Portuguese Eurasian otters [24], which was consistent with the findings of the present study.

The microbiota is often an essential mediator of host nutrient acquisition [25]. In the present study, we found that in the same natural region, the composition of the gut microbiome between Eurasian otters and snub-nosed monkeys was a profound difference. This divergence might be associated with the differences in the dietary structure of these two animals. The intake of bamboo shoots was high in the snub-nosed monkey, and a high content of cyanide compounds has been found in most bamboo shoots [26]. Therefore, the gut microbiomes of bamboo-eating pandas show a high proportion of specific *Proteobacteria* groups (e.g., *Pseudomonadaceae*), which are connected with the cyanide compounds detoxification in bamboo [27]. The snub-nosed monkeys showed the same intestinal flora pattern. The otter diet is various, and prey selection relies on its habitat type [8]; it feeds mainly on fish and sometimes leaves or bark. The fecal microbial alpha diversity of otters was higher than that of snub-nosed monkeys. These results indicated that overall diet type, i.e., carnivory vs. herbivory, plays an evident role in determining the structure of distal gut microbiota.

The diversity of vertebrates’ gut microbial communities is affected by genotype and physiology, called endogenous host factors, and in part by exogenous factors, particularly the environment [6]. Animals have the potential to acquire microorganisms through exposure to natural settings (e.g., soil) and food and water [28]. Further evidence showed that soil biodiversity is interrelated with the gut microbiome [29]. For instance, mice gut microbial diversity was raised through exposure to soil microbes [30]. Snub-nosed monkeys inhabit trees while otters live in water and land, and otters have closer contact with soil than snub-nosed monkeys, and this is why the alpha diversity of otter feces was greater than that of snub-nosed monkeys. Another reason is that animals that rely on a single or few sources of diet typically have lower microbiome diversity [31]. Considering that otters are more omnivorous than monkeys, the relative microbial diversity is greater. Research has shown that three broad factors are generally associated with population heterogeneity in gut microbiota, including (1) host species, behaviors, diets, and environments; (2) biogeography; and (3) host genetic effects [32]. In the present study, the different host species may be the most important cause for the profound difference in the beta diversity (composition) of the gut microbiome of wild foraging monkeys and otters inhabiting the same natural region.

Different dietary structures shape different fatty acid metabolic patterns in the gut [33]. SCFAs, including acetate, propionate, butyrate, isobutyrate, and isovalerate, are the end metabolites of the fermentation of dietary fibers and protein by the gut microbiome, and play a pivotal role in the maintenance of intestinal homeostasis and help maintain energy balance, immune system function, and health [34]. The dietary structure and the complex community of gut microbiota (i.e., its diversity) could influence the changes in SCFA quantity and proportion [35]. In the present study, higher levels of propionic acid in monkeys’ feces than in otters’ feces reflect a higher carbohydrate intake and are related to a higher abundance of *Proteobacteria* [19]. The gut microbiota of otters differed from that of monkeys and thus contributed to higher levels of acetic and isovaleric acids and total SCFAs, which may be related to the higher abundance of *Firmicutes*. Butyric and valeric acid were not detected in this study, possibly because of the time of stool sampling and the volatility of SCFAs [36]. Nevertheless, we recognize a number of limitations in interpreting these results because of the lack of determination of long-chain fatty acid concentrations.

The seasons can also have a certain influence on the gut microbiota of animals. Previous research reported that the gut microbiota of giant pandas exhibited significant seasonal differences [37]. Similar results were also shown in Canadian mink [38], a member of the Mustelidae family similar to the European otter. In this study, sample collection was performed in winter, and animal fecal microorganisms may be different from those in summer and autumn. The main reason is the difference in the abundance of food sources between summer and autumn. However, additional research has shown that age has a greater impact on gut microbiota than seasonality [21]. The next step of our studies should entirely avoid confounding factors such as season to better identify the environmental effect.

Multiple environments, such as soil, water, and air, are all inhabited by microbes [39]. Soil provides space for living and food production for animals [40] while having a profound impact on an animal’s gut microflora [32]. A relationship between the community of gut microbiota and the microbial ecosystem of soil appears possible due to the functional similarities between the two. Here, we match-sampled snub-nosed monkey and Eurasian otter feces with soil, and we analyzed the characteristic variation in host gut microbiota and soil microbiota. So, the first step to comprehending the impact of environmentally acquired microbes on intestinal microbes and population-level differences between hosts’ microbiomes is to investigate the communities of environmental microbes with the associated hosts [6]. However, in this study, only soil samples were studied, and the microorganisms in other habitat elements (water sources, vegetation, etc.) were not studied, which is another limitation of this study.

## 5. Conclusions

In conclusion, this study confirmed that the fecal microbes of Eurasian otters and golden snub-nosed monkeys in a nature reserve are related to the soil microbial communities of their habitats, but they have different bacterial community structures and compositions, and there are different short-chain fatty acid metabolic patterns in the gut of the two animals. These findings may be used as sensitive screening tools to protect critical food resources or habitats for Eurasian otters and golden snub-nosed monkeys.

## Data Availability

The data presented in the present study are deposited in the NCBI repository with accession number PRJNA874883.

## References

[1] Nishida A.H., Ochman H. (2018). Rates of gut microbiome divergence in mammals. Mol. Ecol..

[2] Dudek N.K., Switzer A.D., Costello E.K., Murray M.J., Tomoleoni J.A., Staedler M.M., Tinker M.T., Relman D.A. (2022). Characterizing the oral and distal gut microbiota of the threatened southern sea otter (*Enhydra lutris nereis*) to enhance conservation practice. Conserv. Sci. Pract..

[3] Karmacharya D., Manandhar P., Manandhar S., Sherchan A.M., Sharma A.N., Joshi J., Bista M., Bajracharya S., Awasthi N.P., Sharma N. (2019). Gut microbiota and their putative metabolic functions in fragmented Bengal tiger population of Nepal. PLoS ONE.

[4] Smith C.C., Snowberg L.K., Gregory Caporaso J., Knight R., Bolnick D.I. (2015). Dietary input of microbes and host genetic variation shape among-population differences in stickleback gut microbiota. ISME J..

[5] Frankel J.S., Mallott E.K., Hopper L.M., Ross S.R., Amato K.R. (2019). The effect of captivity on the primate gut microbiome varies with host dietary niche. Am. J. Primatol..

[6] Bornbusch S.L., Greene L.K., Rahobilalaina S., Calkins S., Rothman R.S., Clarke T.A., Lafleur M., Drea C.M. (2022). Gut microbiota of ring-tailed lemurs (*Lemur catta*) vary across natural and captive populations and correlate with environmental microbiota. Anim. Microbiome.

[7] Kim I.S., Sim J.H., Cho J.W., Kim B., Lee Y., Ahn D. (2020). Osteoporosis in an Asian small-clawed otter (*Aonyx cinereus* Illiger, 1815). J. Vet. Med. Sci..

[8] Day C.C., Westover M., Mcmillan B.R. (2015). Seasonal diet of the northern river otter (*Lontra canadensis*): What drives prey selection?. Can. J. Zool..

[9] Qi X.G., Garber P.A., Ji W., Huang Z.P., Huang K., Zhang P., Guo S.T., Wang X.W., He G., Zhang P. (2014). Satellite telemetry and social modeling offer new insights into the origin of primate multilevel societies. Nat. Commun..

[10] Huang Z.P., Bian K., Liu Y., Pan R.L., Qi X.G., Li B.G. (2017). Male dispersal pattern in golden snub-nosed monkey (*Rhinopithecus roxellana*) in Qinling mountains and its conservation implication. Sci. Rep..

[11] Mcfall-Ngai M., Hadfield M.G., Bosch T.C., Carey H.V., Domazet-Lošo T., Douglas A.E., Dubilier N., Eberl G., Fukami T., Gilbert S.F. (2013). Animals in a bacterial world, a new imperative for the life sciences. Proc. Natl. Acad. Sci. USA.

[12] Peng Z., Zhang C., Shen M., Bao H., Hou Z., He S., Hua Y. (2017). Baylisascaris schroederi infection in giant pandas (*Ailuropoda melanoleuca*) in Foping National Nature Reserve, China. J. Wildl. Dis..

[13] Yu Z., Morrison M. (2004). Comparisons of different hypervariable regions of rrs genes for use in fingerprinting of microbial community by PCR-denaturing gradient gel electrophoresis. Appl. Environ. Microbiol..

[14] Li F., Wang Z., Dong C., Li F., Wang W., Yuan Z., Mo F., Weng X. (2017). Rumen bacteria communities and performances of fattening lambs with a lower or greater subacute ruminal acidoss risk. Front. Microbiol..

[15] Sarkar A., Harty S., Johnson K.V., Moeller A.H., Archie E.A., Schell L.D., Carmody R.N., Clutton-Brock T.H., Dunbar R.I.M., Burnet P.W.J. (2020). Microbial transmission in animal social networks and the social microbiome. Nat. Ecol. Evol..

[16] Sharma A.K., Petrzelkova K., Pafco B., Jost Robinson C.A., Fuh T., Wilson B.A., Stumpf R.M., Torralba M.G., Blekhman R., White B. (2020). Traditional human populations and nonhuman primates show parallel gut microbiome adaptations to analogous ecological conditions. mSystems.

[17] Clayton J.B., Gomez A., Amato K., Knights D., Travis D.A., Blekhman R., Knight R., Leigh S., Stumpf R., Wolf T. (2020). The gut microbiome of nonhuman primates: Lessons in ecology and evolution. Am. J. Primatol..

[18] Moeller A.H., Li Y., Mpoudi Ngole E., Ahuka-Mundeke S., Lonsdorf E.V., Pusey A.E., Peeters M., Hahn B.H., Ochman H. (2014). Rapid changes in the gut microbiome during human evolution. Proc. Natl. Acad. Sci. USA.

[19] Xia W., Liu G., Wang D., Chen H., Zhu L., Li D. (2022). Functional convergence of Yunnan snub-nosed monkey and bamboo-eating panda gut microbiomes revealing the driving by dietary flexibility on mammal gut microbiome. Comput. Struct. Biotechnol. J..

[20] Guo G., Eccles K.M., Mcmillan M., Thomas P.J., Chan H.M., Poulain A.J. (2020). The gut microbial community structure of the north American river otter (*Lontra canadensis*) in the Alberta oil sands region in Canada: Relationship with local environmental variables and metal body burden. Environ. Toxicol. Chem..

[21] Okamoto Y., Ichinohe N., Woo C., Han S.Y., Kim H.H., Ito S., Nakamura C., Kumura J., Nagaoka K., Yamamoto N. (2021). Contrasting gut microbiota in captive Eurasian otters (*Lutra lutra*) by age. Arch. Microbiol..

[22] Chen L., Liu M., Zhu J., Gao Y., Sha W., Ding H., Jiang W., Wu S. (2020). Age, gender, and feeding environment influence fecal microbial diversity in spotted hyenas (*Crocuta crocuta*). Curr. Microbiol..

[23] He F., Liu D., Zhai J., Zhang L., Ma Y., Xu Y., Rong K., Ma J. (2018). Metagenomic analysis revealed the effects of goat milk feeding and breast feeding on the gut microbiome of Amur tiger cubs. Biochem. Biophys. Res. Commun..

[24] Oliveira M., Sales-Luís T., Duarte A., Nunes S.F., Carneiro C., Tenreiro T., Tenreiro R., Santos-Reis M., Tavares L., Vilela C.L. (2008). First assessment of microbial diversity in faecal microflora of Eurasian otter (*Lutra lutra* Linnaeus, 1758) in Portugal. Eur. J. Wildl. Res..

[25] Amato K.R., Yeoman C.J., Cerda G., Schmitt C.A., Cramer J.D., Miller M.E., Gomez A., Turner T.R., Wilson B.A., Stumpf R.M. (2015). Variable responses of human and non-human primate gut microbiomes to a Western diet. Microbiome.

[26] Sang A.G.P., Guharat S., Wananukul W. (2011). A mass cyanide poisoning from pickling bamboo shoots. Clin. Toxicol..

[27] Zhang Z., Hu T., Lu G., Zhu L. (2020). Lessons from bamboo-eating pandas and their gut microbiome: Gut microbiome flow and applications. Evol. Appl..

[28] Selway C.A., Mills J.G., Weinstein P., Skelly C., Yadav S., Lowe A., Breed M.F., Weyrich L.S. (2020). Transfer of environmental microbes to the skin and respiratory tract of humans after urban green space exposure. Environ. Int..

[29] Blum W.E.H., Zechmeister-Boltenstern S., Keiblinger K.M. (2019). Does soil contribute to the human gut microbiome?. Microorganisms.

[30] Zhou D., Zhang H., Bai Z., Zhang A., Bai F., Luo X., Hou Y., Ding X., Sun B., Sun X. (2016). Exposure to soil, house dust and decaying plants increases gut microbial diversity and decreases serum immunoglobulin E levels in BALB/c mice. Environ. Microbiol..

[31] Senghor B., Sokhna C., Ruimy R., Lagier J.C. (2018). Gut microbiota diversity according to dietary habits and geographical provenance. Hum. Microb. J..

[32] Grieneisen L.E., Charpentier M.J.E., Alberts S.C., Blekhman R., Bradburd G., Tung J., Archie E.A. (2019). Genes, geology and germs: Gut microbiota across a primate hybrid zone are explained by site soil properties, not host species. Proc. Biol. Sci..

[33] Loo Y.T., Howell K., Chan M., Zhang P., Ng K. (2020). Modulation of the human gut microbiota by phenolics and phenolic fiber-rich foods. Compr. Rev. Food Sci. Food Saf..

[34] Puddu A., Sanguineti R., Montecucco F., Viviani G.L. (2014). Evidence for the gut microbiota short-chain fatty acids as key pathophysiological molecules improving diabetes. Mediat. Inflamm..

[35] Dowd S.E., Callaway T.R., Wolcott R.D., Sun Y., Mckeehan T., Hagevoort R.G., Edrington T.S. (2008). Evaluation of the bacterial diversity in the feces of cattle using 16S rDNA bacterial tag-encoded FLX amplicon pyrosequencing (bTEFAP). BMC Microbiol..

[36] Furuhashi T., Sugitate K., Nakai T., Jikumaru Y., Ishihara G. (2018). Rapid profiling method for mammalian feces short chain fatty acids by GC-MS. Anal. Biochem..

[37] Guo W., Chen Y., Wang C., Ning R., Zeng B., Tang J., Li C., Zhang M., Li Y., Ni Q. (2020). The carnivorous digestive system and bamboo diet of giant pandas may shape their low gut bacterial diversity. Conserv. Physiol..

[38] Compo N.R., Gomez D.E., Tapscott B., Weese J.S., Turner P.V. (2018). Fecal bacterial microbiota of Canadian commercial mink (Neovison vison): Yearly, life stage, and seasonal comparisons. PLoS ONE.

[39] Yang J., Yang Y., Ishii M., Nagata M., Aw W., Obana N., Tomita M., Nomura N., Fukuda S. (2020). Does the gut microbiota modulate host physiology through polymicrobial biofilms?. Microbes Environ..

[40] Brevik E.C., Pereg L. (2017). History of soils in relation to animal and human health. The Nexus of Soils, Plants, Animals and Human Hearth.

